# A model of spatio-temporal regulation within biomaterials using DNA reaction–diffusion waveguides

**DOI:** 10.1098/rsos.220200

**Published:** 2022-08-24

**Authors:** Phillip J. Dorsey, Dominic Scalise, Rebecca Schulman

**Affiliations:** ^1^ Department of Chemical and Biomolecular Engineering, Johns Hopkins University, 3400 North Charles Street, Baltimore, MD 21218, USA; ^2^ Department of Computer Science, Johns Hopkins University, 3400 North Charles Street, Baltimore, MD 21218, USA

**Keywords:** chemical reaction networks, DNA nanotechnology, reaction–diffusion systems

## Abstract

In multi-cellular organisms, cells and tissues coordinate biochemical signal propagation across length scales spanning micrometres to metres. Designing synthetic materials with similar capacities for coordinated signal propagation could allow these systems to adaptively regulate themselves across space and over time. Here, we combine ideas from cell signalling and electronic circuitry to propose a biochemical waveguide that transmits information in the form of a concentration of a DNA species on a directed path. The waveguide could be seamlessly integrated into a soft material because there is virtually no difference between the chemical or physical properties of the waveguide and the material it is embedded within. We propose the design of DNA strand displacement reactions to construct the system and, using reaction–diffusion models, identify kinetic and diffusive parameters that enable super-diffusive transport of DNA species via autocatalysis. Finally, to support experimental waveguide implementation, we propose a sink reaction and spatially inhomogeneous DNA concentrations that could mitigate the spurious amplification of an autocatalyst within the waveguide, allowing for controlled waveguide triggering. Chemical waveguides could facilitate the design of synthetic biomaterials with distributed sensing machinery integrated throughout their structure and enable coordinated self-regulating programmes triggered by changing environmental conditions.

## 1. Introduction

The biomolecular components of cells are powerful computational tools. They serve as exquisite detectors of signalling molecules [1,2] and metabolites [3,4], orchestrate multi-step chemical synthesis and catalysis, and self-assemble nanostructures [5] or materials with unique structural properties [6] and geometry [7]. Synthetic biomolecular sensors can detect concentrations of drugs in the blood in real time [8,9], approaching the sensitivity with which cells detect substances. Similarly, engineered enzyme cascades can orchestrate multi-stage chemical reactions [10], biomolecular assemblies can template electronic devices [11,12], and therapeutics can sense local conditions and dispense medication at the right time [13–15]. Recently, engineers and nanotechnologists have sought to design synthetic materials capable of sensing, integrating and transmitting spatial information in processes similar to the functions performed by vascularized tissues. Microfabricated systems composed of fluidic or pneumatic vasculature have been designed to coordinate and direct delivery of fuel or nutrients to various locations in soft polymer substrates [16] in order to control actuation and growth and migration of cells in tissue scaffolds [17]. However, fluidic control mechanisms present several challenges for designing triggerable sensing, communication and computation in material systems. These systems often require tethers to external power sources or fuel depots that are difficult to integrate within the structure of the material.

Here, we use molecular programming concepts from synthetic biology and DNA nanotechnology to leverage the dynamics of nonlinear reaction networks coupled to diffusive transport of molecular species to show how to achieve super-diffusive transport of chemical signals. Reaction–diffusion waveguides or wires consist of a region within a hydrogel substrate that acts as an excitable medium, where an autocatalytic reaction propagates spatially in the form of a travelling wavefront. Multiple wires could be integrated within a substrate and insulated from one another using competitive reactions that restrict autocatalytic reactions to the specific paths of the different wires. Chemical reactions generally take seconds to hours to reach completion and require minimally nano- to microlitre volumes to ensure deterministic behaviour. As such, chemical wires are not intended to compete with electronic wires in terms of speed or reliability. Instead, the biochemical waveguides we develop are designed to enable the coordination of biomolecular sensors, polymer actuators, biomaterials and soft robots in millimetre-scale soft materials without the need for electronics.

The idea of controlling information propagation through chemical reactions and directing information processing stems from Alan Turing's foundational research regarding the origins of pattern formation during morphogenesis; this work described how periodic spatial patterns of chemical species could arise from transient fluctuations within an initially homogeneous system [18]. Experimental implementations of the Belousov–Zhabotinsky reaction–diffusion system (specifically aerosol OT microemulsion and chlorite–iodide–malonic acid reaction systems) have been demonstrated as mechanisms for propagating chemical species across space [19,20]. Recently, the growing research field of DNA nanotechnology has provided new routes of material programming, enabling the design of experimental oscillators and amplifiers composed of biomolecular components capable of interfacing with biological systems.

Synthetic biomolecular devices have been developed to release or respond to nucleic acid (DNA or RNA) signals of 20–100 bases in length. These signals can start or stop molecular machines [21] or catalysis [22], and direct hydrogel [23,24] or nanostructure [25,26] self-assembly. Nucleic acid signals can also be released by aptamer or antibody sensors [27,28]. Molecular circuits can represent information as concentrations of nucleic acids in solution. Similar to electronic circuits, molecular circuits perform complex computation by emulating the functions of Boolean logic gates to conduct mathematical operations [29] and can implement neural networks for pattern recognition [30]. These circuits have been used to classify or execute logic operations on multiple nucleic acid signal inputs, act as memory latches, or direct oscillatory cycles of signal activity [29,31,32]. Enzymatic mechanisms may also be coupled with nucleic acids to initiate and regulate spatio-temporal changes in a system. For example, Bauer *et al*. demonstrated the formation of travelling waves of RNA *in vitro* generated from the Q*β* viral enzyme and a self-replicating RNA species in a liquid filled capillary [33]. Zadorin *et al*. used a polymerase–exonuclease–nickase enzyme circuit with a template DNA duplex to produce a travelling wavefront within a buffer-filled polystyrene channel [34]. Similarly, Zambrano *et al*. implemented an enzymatic predator–prey reaction network within a microfluidic network to compute the shortest distance within the network from entrance to exit [35]. Here, we explore the design of non-enzymatic chemical reaction networks for propagating molecular signals. Temperature-dependent and batch-dependent variation in catalytic activity is often observed with enzymatic systems, which can yield varying stimuli-responsiveness between otherwise identical architectures [36,37]. These challenges could be avoided by using enzyme-free chemical reaction networks, which facilitate scalable and predictable design of molecular architectures where such sensitivities are unacceptable, and aid construction of more complex DNA-programmable systems capable of executing spatial computation.

To this end, Chatterjee *et al*. [38] implemented surface-immobilized DNA complexes to catalytically exchange signals via strand displacement between DNA dominoes over nanometre length scales. Joesaar *et al*. [39] developed a sender–receiver network consisting of DNA-encapsulated proteinosomes organized into regularly spaced arrays within a microfluidic trapping device as a means of exchanging chemical signals spatially between specific locations via diffusing DNA species. These microcapsules possessed DNA-permeable BSA-NH_2_/PNIPAAM cross-linked membranes and contained non-enzymatic catalytic DNA strand displacement circuits; when triggered by an externally supplied DNA signal, the microcapsule array executed distributed logic operations by exchanging diffusing DNA signals between sender and receiver microcapsules. Yang *et al*. [40] built upon this work by incorporating a photo-triggerable DNA strand displacement latch within sender microcapsules to enable distributed logical operations triggered through spatial activation with a laser. These studies are excellent models for how synthetic DNA-based systems might enable distributed communication spatially. However, the design of triggerable non-enzymatic DNA reaction–diffusion systems capable of transmitting chemical signals spatially as part of an integrated biomaterial substrate and the identification of experimentally attainable kinetic and diffusive parameters for such systems to achieve a rate of signalling faster than that provided by diffusion alone remain understudied.

Synthetic biologists and molecular programmers have also sought to understand how nonlinear chemical behaviours might be adapted, using DNA, to program excitatory responses in systems for signal amplification and information exchange in well-mixed conditions. Such nonlinear dynamics, often consisting of chemical oscillators or amplifiers, commonly incorporate autocatalysis as a mechanism of amplification [41–43]. Here, we extend these analyses to characterize how such systems could function to enable directed spatial propagation of a DNA input as a reaction–diffusion system, and more specifically, how a strand displacement amplification network that we adapted from Zhang *et al*. [44] could achieve this functionality. Gines *et al*., Lysne *et al*. and Srinivas *et al*. previously investigated thresholded autocatalytic DNA-based reaction networks in well-mixed systems [41–43]. Gines *et al*. implemented an enzymatic sink reaction in which an exonuclease quenched spurious amplification during microRNA detection [41]. Srinivas *et al*. designed a strand displacement thresholding reaction that consumed autocatalyst species produced by parasitic reactions in an oscillatory strand displacement reaction network [43]. Lysne *et al*. implemented a leak-mitigation strategy by designing small interfering domains that hybridized to sections of a fuel oligonucleotide to sterically hinder a leak reaction occurring in the well-mixed Zhang autocatalytic strand displacement amplifier [42].

**Figure 1.**
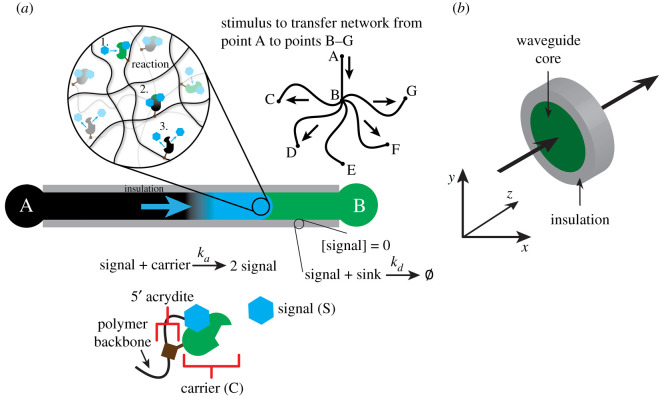
Design and function of a reaction–diffusion waveguide in a hydrogel. (*a*) A chemical wave of signal is propagated between points A and B via an autocatalytic reaction that makes copies of signal from a carrier species that is cross-linked to the hydrogel network. Such a system could be used to route chemical signals simultaneously between multiple points in space: (1) signal reacts with patterned carrier, (2) carrier transitions into its release state and (3) carrier releases two signal molecules. (*b*) Schematic cross-section of a three-dimensional waveguide showing its core where autocatalysis occurs and the insulation surrounding it which prevents signal from diffusing from the waveguide.

Here, we propose a means of biomolecular signal propagation within soft materials: an insulated reaction–diffusion waveguide that could enable super-diffusive transport over distances of hundreds of micrometres to millimetres (figure 1*a*). The waveguide insulation prevents propagated signals from spreading beyond the waveguide and acts as a chemical sink. By simultaneously directing and restricting transport of a chemical species, this design could accommodate integration of multiple waveguides to transmit multiple signals in parallel or propagate several different molecular inputs within a single substrate without triggering crosstalk. To develop a design for this waveguide, we first determine what rates of spatial propagation could be achieved by autocatalytic waves using an abstract autocatalytic chemical reaction network composed of a series of coupled bimolecular reactions within a reaction–diffusion medium. We then design a thresholding and amplification quenching strategy to enable insulation of waveguides and simultaneously prevent spurious activation by undesired leak reactions between different DNA species, similar to the damping strategy originally postulated by Zhang & Seelig [45] and the thresholding reaction implemented by Srinivas *et al*. [43]. Finally, we implement the abstract enzyme-free autocatalysis reaction network and thresholding reaction as a DNA strand displacement reaction network designed for use in spatial signal propagation and show by measuring its rates in experiments, that this reaction scheme could be used to operate a triggerable autocatalytic waveguide. Together these results suggest a roadmap for the design and implementation of waveguides as means of accelerated molecular communication.

## 2. Material and methods

### 2.1. Reaction–diffusion waveguide model

We constructed models of the reaction–diffusion waveguide using Comsol Multiphysics finite-element analysis software. Specific details regarding the models' geometry, initial conditions, reactions and diffusion coefficients are provided in the electronic supplementary material, S3, and Results section.

### 2.2. DNA strand displacement reactions

DNA strands were purchased from Integrated DNA Technologies (Coralville, IA). The annealing and purification protocol for double-stranded DNA complexes as well as the protocol for reaction progress monitoring using quantitative PCR are provided in the electronic supplementary material, S1. Curve fitting analysis to determine reaction rate constants is discussed in the electronic supplementary material, S4.

## 3. Results

### 3.1. Autocatalytic amplification enables super-diffusive transport of biochemical species in a reaction–diffusion waveguide model

**Figure 2.**
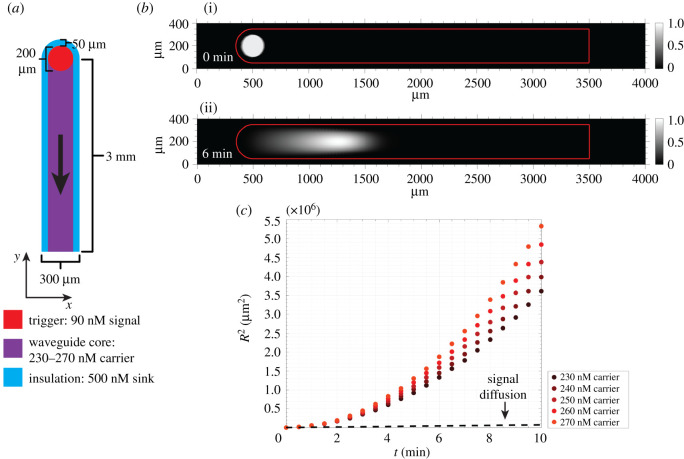
An idealized model of a reaction–diffusion waveguide. (*a*) Geometry of the waveguide model and initial concentrations used for the amplification reaction. (*b*) (i) The initial distribution of signal before triggering and (ii) the distribution of signal after 6 min of wavefront propagation down the waveguide. Both surface plots show concentrations normalized to maximum signal concentration; the red frame indicates the boundaries of the waveguide. (*c*) Squared wavefront displacement versus time across five different carrier concentrations. The dashed black line shows the squared displacement of a 42 nucleotide-sized single-stranded DNA molecule over time resulting from diffusion alone.

A reaction–diffusion waveguide consisted of a path within a hydrogel where specific DNA molecules were conjugated to the hydrogel's polymer network.^1^ We modelled waveguide behaviour using a two-dimensional simulation where the waveguide path consisted of a two-dimensional area (figure 2*a*). The molecules along the path were the reactants and fuel consumed in an autocatalytic reaction. Another region of the hydrogel in which a different set of molecules was conjugated, termed as the insulation, lined the edges of the waveguide. The insulation contained a high concentration of a DNA species conjugated to the network that acted to prevent the wave propagating along the waveguide from extending beyond its boundaries. The following abstract reactions were propagated along the waveguide:

$$\mathrm{Signal}\;+\mathrm{Carrier}\overset{k_{a}}{⟶}2\;\mathrm{Signal}\;\;(\mathrm{Reaction}\;1)$$

and

$$\mathrm{Signal}+\mathrm{Sink}\overset{k_{d}}{⟶}\;\phi \;\;(\mathrm{Reaction}\;2)$$

The signal species served as a trigger for the autocatalytic reaction cascade and the operation of the waveguide involved the spatial propagation of a high concentration of signal. Our model included a site for triggering wave propagation of signal through the waveguide (location A in figure 1*a*). When signal was present at location A, it could diffuse into the core wire domain and react with carrier in the waveguide's path to produce more signal. The model of this process was designed to predict experiments that would be used to characterize a waveguide's function. In such experiments, location A would contain signal that is covalently linked to the hydrogel and thus could not diffuse into the waveguide. Shining UV light or another spatially modulated stimulus on location A would cleave the bonds connecting signal to location A, allowing signal to diffuse and trigger the waveguide's operation. The waveguide propagates a chemical wave when signal is added, because signal can react with carrier autocatalytically to produce two molecules of signal (Reaction 1). Produced signal can then diffuse and react with more carrier and thus generate more signal. To make it possible to remove signal from the waveguide after it was produced, we added Reaction 2, in which a sink molecule reacts rapidly with signal to convert it into waste. Carrier and sink were immobilized within the waveguide core.

The simplest method of transmitting information in the form of a particular molecular species between two points in space is to allow molecules to passively diffuse from a region of higher concentration to one of lower concentration. The characteristic time for this process to occur over a distance *L* scales with *O*(*L*^2^) according to Fick's second law.

Coupling a reaction to this diffusive process could decrease the time over which information is transmitted. Specifically, if a diffusing molecule, signal, reacts with a patterned path of carrier molecules to create copies of itself (as in Reaction 1), then signal will form a moving wave in which signal diffuses and also amplifies itself. With certain rates of reaction and diffusion, this process can produce a stable travelling wave. Such a wave forms when the autocatalytic reactions change the shape and magnitude of the Laplacian at the leading edge of the wavefront in such a way as to yield a linear rate of displacement with respect to time (assuming a constant concentration of carrier along the waveguide path). The existence of a stable asymptotic travelling wavefront and the analytical relationship between reaction rate, diffusivity and wave velocity can be elucidated by adapting the Fisher–Kolomogorov–Petrovsky–Piskunov (FKPP) treatment [46–48] of a one-dimensional reaction–diffusion process for the autocatalytic network described above. The full derivation of the equation is provided in electronic supplementary material, section S5. The final expression for the minimum rate of displacement for the autocatalytic front in the presence of sink is

3.1 $$v_{\min}=2\sqrt{D_{Sg}(k_{a}C_{\max}-k_{d}Sk_{\max})},$$

where *D_Sg_* is the diffusion coefficient of the signal, *C*_max_ and *Sk*_max_ is the initial concentration of carrier and sink patterned within the core waveguide path, and *k_a_* and *k_d_* are the bimolecular rate constants for the abstract amplification and sink reactions, respectively.

A key result of the FKPP analysis of the abstract amplification scheme is that the square of the effective change in displacement of the autocatalyst species in one-dimensional space (*δL*^2^) over time (*δt*) is proportional to the square of the change in time, *δL*^2^ ∝ *δt*^2^*4*D_Sg_*; this results in a power law dependence between *δL*^2^ and *δt* and a super-diffusive regime of signal transport where the exponent of *δt* is greater than 1. For diffusion in the absence of any reaction, *δL*^2^ ∝ 2*D_Sg_δt*, which yields a linear relationship between the square of the displacement and time.

To verify that this idealized reaction–diffusion amplifier could achieve super-diffusive transport of an autocatalyst species, we simulated its behaviour in a reaction–diffusion model. The model was implemented using Comsol Multiphysics (Transport of Dilute Species physics node). The waveguide was 3000 µm long and 300 µm wide (figure 2*a*). The insulation surrounding the edges of the waveguide was 50 µm wide. In the model, the domain holding the initial signal stimulus to trigger the system at one end of the waveguide (location A in figure 1) consisted of a 100 µm radius circle.

Our first analysis modelled abstract reactions 1 and 2 and considered wave propagation when sink was patterned within the insulation but not within the core waveguide path where carrier was localized. The primary goal of these simulations was to determine whether idealized reactions 1 and 2 could form a stable travelling wave using net reaction rates that could be experimentally implemented for a toehold-mediated DNA strand displacement reaction network. A secondary goal was to determine the form and speed of a travelling wave using an experimentally measured diffusion coefficient for short DNA molecules in poly(ethylene glycol) diacrylate hydrogels in order to show that these biomaterials could be used as substrate for the reaction network. The bimolecular rate constants for the modelled reactions were selected using measured rate constants for toehold-mediated strand displacement reactions at 25°C in standard buffer conditions [49]. Strand displacement toeholds typically range in size from 0 to 7 nucleotides. Above toeholds of 7 nucleotides, the magnitude of the bimolecular rate constant saturates. Therefore, in order to design an amplifier that reacted at the fastest rate possible, we designed these reactions to occur with rate constants at the upper end of this scale. Specifically, we chose *k_a_* to be 2 × 10^5^ M^−1^ s^−1^, which is the order of magnitude for a bimolecular rate constant in a six-nucleotide (nt) toehold strand displacement reaction. We set *k_d_* to be 4 × 10^6^ M^−1^ s^−1^, which is the order of magnitude for a standard 7-nt toehold reaction [49]. To ensure that the sink reaction could successfully perform its function of restricting amplification to the waveguide, we set the rate constant for its reaction with signal to be an order of magnitude higher than the rate constant for the carrier and signal reaction. Signal was assigned a diffusion coefficient of 60 µm^2^ s^−1^, a typical value for the diffusivity of a 42-nt single-stranded (ss) DNA oligonucleotide in poly(ethylene glycol) diacrylate (*M*_n_ = 575) hydrogels [50]. Sink and carrier were immobilized within the waveguide insulation and waveguide core respectively (i.e. their diffusion constants were 0). The concentration of sink in the insulation was 500 nM. The initial concentration of signal within the triggered domain was 90 nM. The initial concentration of carrier in the waveguide varied between 230 nM and 270 nM by steps of 10 nM in each simulation.

Across all carrier conditions tested, we observed the formation of a stable signal wavefront that travelled along the waveguide as it consumed carrier (figure 2*b*,*c*). Additionally, the wave was constrained to the waveguide; it did not spread beyond the insulation. We calculated the displacement of the wavefront in the centre axis of the waveguide over time (figure 2*c*) and observed that the square of the displacement, *R*^2^, was proportional to *t^α^*, with *α* > 1, indicating that the idealized system had achieved super-diffusive transport of signal. The dashed black line in figure 2*c* indicates the square of the displacement resulting from simple diffusion of a DNA oligonucleotide in one dimension.

**Figure 3.**
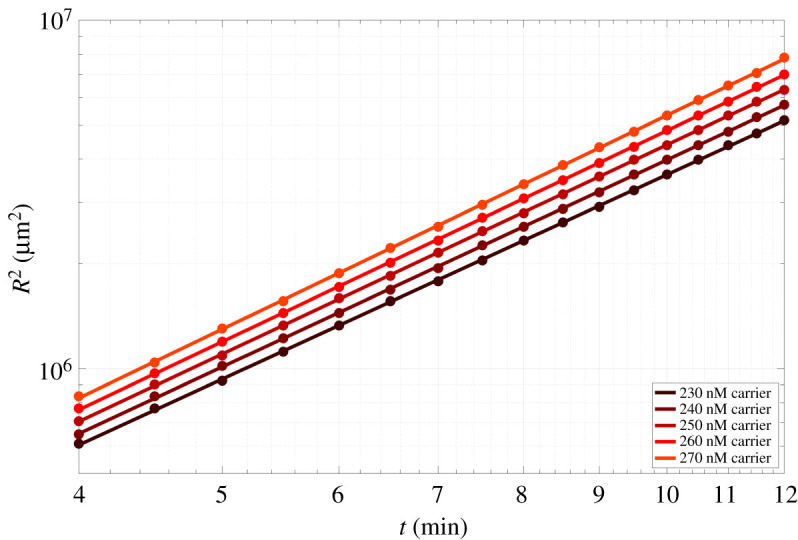
Log-scaled plot of the squared wavefront displacement (*R*^2^) versus time of the idealized waveguide with no sink present in the core path for varying carrier concentrations. Dots indicate the squared displacement measured in simulations. Lines are the linear least-squares fit of *R*^2^ versus time. A linear relationship exists between log(*R*^2^) and log(time) at these values.

While *R*^2^ for the reaction network grows exponentially with time, *R*^2^ in the case of simple diffusion grows linearly with time. Plots of *R*^2^ versus time on a logarithmic plot yielded straight lines across all carrier concentrations (figure 3), where the slope of the line was *α*. Across all carrier concentrations, the average value of *α* calculated from the least-squares fits of *R*^2^ versus time shown in figure 3 was 2.00 ± 0.01 (95% confidence interval). The length of the region where signal was present grew over time because of an increase in signal concentration down the length of waveguide (figure 2*b*). Additionally, we observed that at each individual timepoint, *R*^2^ increased linearly with carrier concentration, which was predicted by FKPP analysis (electronic supplementary material, S5).

Having established that the waveguide design could reliably propagate a spatio-temporal wave using known experimental ranges of parameters for DNA strand displacement reactions and diffusion coefficients, we then tested whether it was possible to form a stable travelling wave with sink patterned within the waveguide core and in the insulation. The inclusion of sink within the waveguide path provides a key function. Sink can react with signal at a faster rate than carrier, so sink serves as a threshold that can protect the waveguide from spurious activation by leak reactions that produce signal.

**Figure 4.**
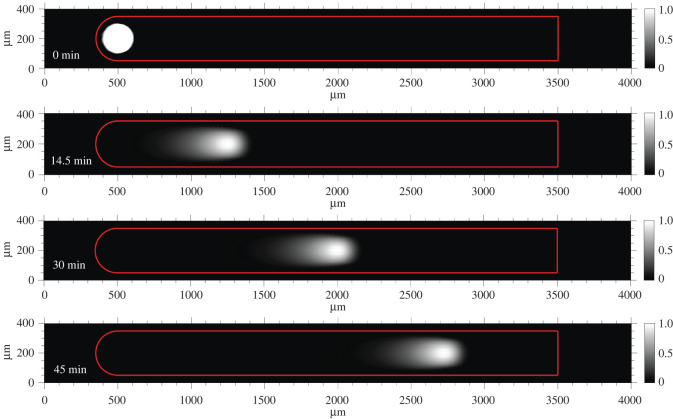
Wave propagation on reaction–diffusion waveguide with 35 nM sink patterned in the wire core. Spatial propagation of the autocatalytic wavefront over time. Here, signal trailing the wavefront is eventually degraded. Surface plots are non-dimensionalized by the maximum concentration of signal within the stable travelling wavefront. The red frame indicates the boundaries of the waveguide.

**Figure 5.**
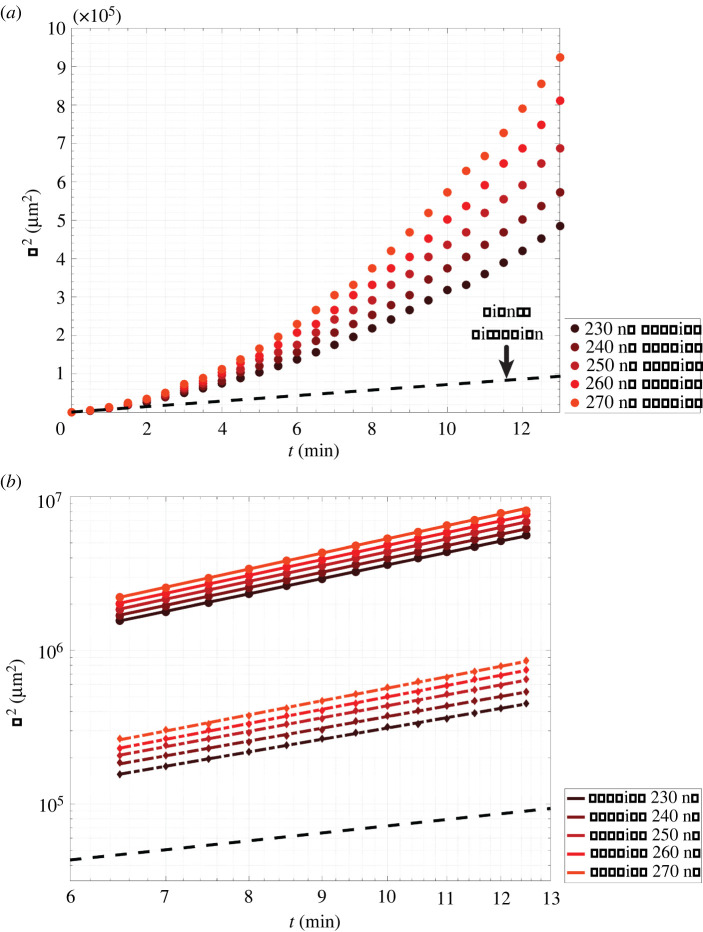
Idealized autocatalytic wavefront propagation in the presence of 35 nM sink. (*a*) Square of the wavefront displacement, *R*^2^, versus time. (*b*) Comparison of *R*^2^ without sink (circles are results of PDE reaction–diffusion model and solid lines are the line of best fit for *R*^2^ versus time) and with 35 nM sink (diamonds are the results of the PDE reaction–diffusion model and dashed lines are the line of best fit for *R*^2^ versus time) patterned in the waveguide core. Black dashed lines in (*a*) and (*b*) indicate *R*^2^ for simple diffusion of a 42 nucleotide DNA molecule.

The simulation used all of the existing conditions described previously and included 35 nM of sink sequestered within the waveguide core. We observed the formation of a stable travelling wave in which signal was degraded into waste at the trailing edge (figure 4). We again observed a nonlinear dependence of *R*^2^ with time (figure 5*a*). Logarithmic plots of *R*^2^ against time yielded a linear relationship across all carrier concentrations (figure 5*b*, diamonds and dashed lines). In the presence of 35 nM sink, the average *α* calculated by the line of best fit of *R*^2^ versus time across all carrier concentrations and plotted timepoints was 1.71 ± 0.04 (95% confidence interval), indicating that the dynamics of wavefront displacement were still in between the thresholds of directed transport (*α* = 2) and super-diffusive transport (*α* > 1). However, the presence of the sink did significantly impede signal propagation: when 35 nM sink was in the waveguide core, the *R*^2^ values at each timepoint were 10-fold smaller than in the absence of sink. Compared to the 0 nM sink condition, the 35 nM sink condition reduced the amount of signal produced by the wavefront which reduced the wave's relative amplitude, width and speed (electronic supplementary material, figure S9). When the autocatalytic rate constant was reduced from 2 × 10^5^ M^−1^ s^−1^ to 5 × 10^4^ M^−1^ s^−1^ (corresponding to a reduction in toehold size from six to five nucleotides), the waveguide was unable to produce a wave. However, increasing the rate of amplification by raising the concentration of carrier to 780 nM recovered wave generation (electronic supplementary material, figure S4).

### 3.2. Designing DNA reactions for synthesizing a waveguide: adding a thresholding reaction to an autocatalytic strand displacement amplifier mitigates the amplifier's spurious activation

**Figure 6.**
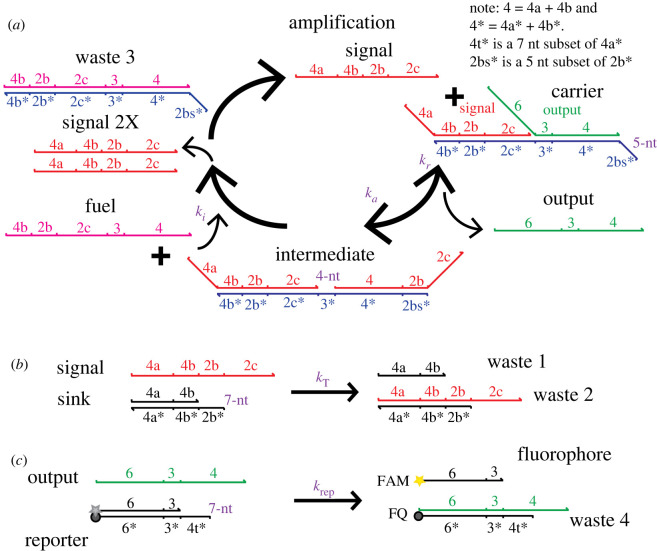
Autocatalytic amplification [44] reactions with thresholding. (*a*) Autocatalysis module. (*b*) Thresholding reaction module. (*c*) Fluorescence reporting reaction module for optical detection.

We next sought to design a chemical reaction network that emulates reactions 1 and 2 using DNA reactions. Designing molecules that react precisely as reactions 1 and 2 was infeasible because the autocatalytic step comprising Reaction 1 cannot be implemented as a single-step bimolecular reaction. Reaction 1 was therefore broken into a series of bimolecular strand displacement reactions involving the carrier species (figure 6*a*). Minimizing the number and complexity of the reactions required to recapitulate the autocatalytic step provided multiple potential benefits: (i) this limited the number of potential leak reactions that might occur during waveguide operation or when constructing the system and (ii) simplified wire construction with top-down methods such as micro-moulding or photolithography. To understand whether DNA strand displacement reactions could be used to build a DNA waveguide based on these criteria, we adopted an autocatalytic DNA strand displacement amplifier previously designed by Zhang *et al*. [44] to add a thresholding reaction (figure 6*b*). We then asked whether the resulting reactions could be used to operate a waveguide by executing reactions 1 and 2.

The strand displacement implementation of the autocatalytic core of the waveguide consisted of the following reactions: signal, a single-stranded (ss) autocatalytic DNA species, first reacted with carrier, a complex consisting of signal and output strands hybridized to a third strand (figure 6*a*). After signal hybridized to the 5-nt length toehold of this complex, it branch migrated to displace output, forming intermediate, a three-strand duplex possessing an exposed toehold (denoted 3*) that fuel could bind to. The reaction between signal and carrier was reversible because output could also rehybridize to intermediate and initiate the reverse reaction.

Fuel and intermediate complex then reacted through a 4-nt toehold and released two signal strands, each of which then reacted with more carrier species. Importantly, a large reservoir of fuel existed within the system, which drove the reaction forward.

To incorporate the insulating and thresholding functions that are key for waveguide function, we created an irreversible thresholding reaction between signal and a sink duplex (figure 6*b*): signal hybridized to sink through a 7-nt toehold. We used a reporting reaction to monitor the progress of the reaction network (figure 6*c*). The output strand produced during the amplification cycle reacted with a reporter duplex composed of a terminal fluorophore–quencher pair to displace its cover fluorophore strand, enabling optical measurement of the circuit's reaction progress using quantitative PCR or fluorescence microscopy.

**Figure 7.**
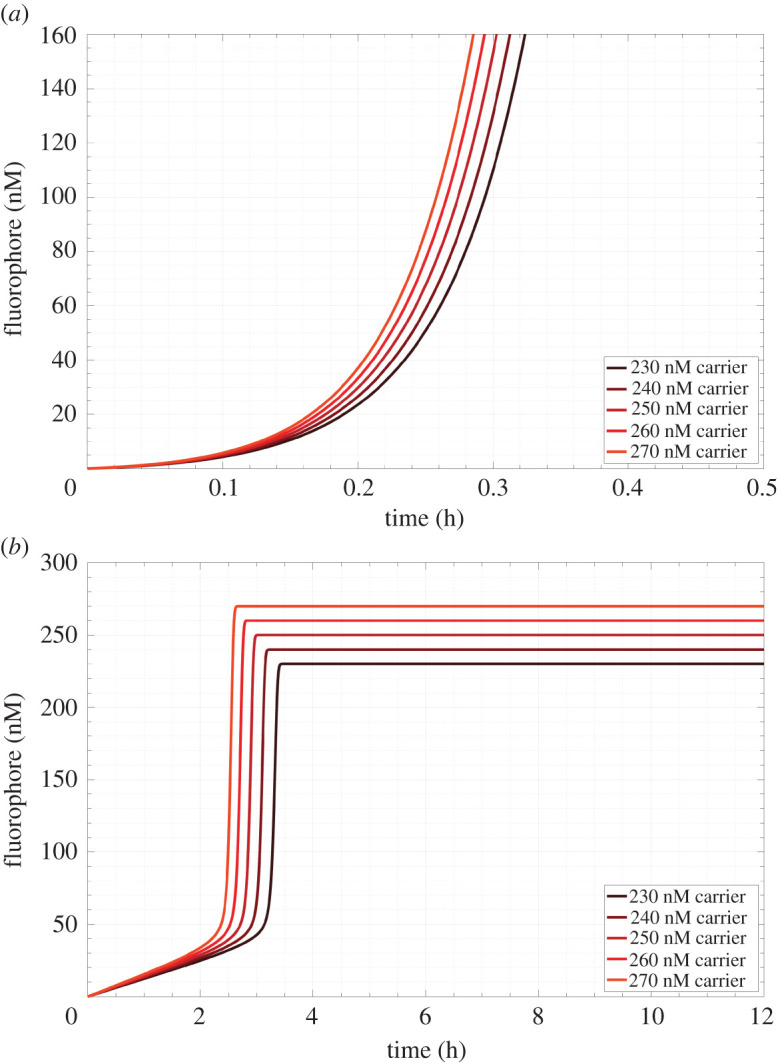
Predictions of a well-mixed reaction model of thresholded autocatalysis. (*a*) The kinetics of amplification when the carrier's toehold is six nucleotides. (*b*) The kinetics of amplification when the carrier's toehold is five nucleotides.

Signal was produced spuriously by the reaction of fuel and carrier [42,44] via base dehybridization at the carrier duplex terminus and at the nick in the duplex between bound output and signal strands. This caused untriggered amplification in the absence of signal and presented a serious challenge for the use of the amplifier in a spatial system where reactants would be incubated with one another over potentially several hours. We developed a model of the full reaction network in well-mixed conditions to determine the timescale of spurious amplification over a range of concentration conditions. The model was composed of a system of ordinary differential equations (ODEs) and used measured values for the strand displacement reaction rate constants [44,49] listed in figure 6 and for the carrier–fuel leak reaction (electronic supplementary material, S6). We observed that for 230–270 nM carrier incubated with 500 nM fuel and 50 nM sink the circuit rapidly entered the growth phase of its sigmoidal activation curve after only 12 min (figure 7*a*). In the absence of any protection chemistry for the carrier or fuel species to prevent leakage upon mixing, such a short timescale of activation provided no feasible way for experimental construction of a hydrogel waveguide in a laboratory setting where experimental set-up times range from tens of minutes to several hours.

To make it possible to build a waveguide, we sought to adapt these reactions in order to increase the time before the autocatalytic reaction proceeded spontaneously (i.e. the lag time of the circuit). We considered several strategies to increase the lag phase of the circuit; each entailed performance tradeoffs. Increasing the rate of the threshold reaction by increasing the sink concentration delays amplification at the cost of raising the threshold concentration of signal needed to trigger the waveguide. Lowering the rate of amplification by either decreasing the concentrations of carrier and fuel or decreasing the rate constant for the amplification reaction would also prolong the lag phase of the reaction at the cost of slowing triggered waveguide operation. We chose to decrease the rate of amplification by shortening the carrier toehold involved in the reaction between signal and carrier from six to five nucleotides, thereby decreasing the rate constant for the reaction by about a factor of 10. With this modification, in the presence of 50 nM sink, 500 nM fuel, and varying concentrations of carrier, the model predicted that the time before the concentration of signal increased superlinearly was roughly 2.1–2.4 h (figure 7*b*). The linear increase in fluorophore concentration was associated with the production of output from the leak reaction between carrier and fuel before the onset of autocatalysis. If sink is present, no autocatalysis occurs and the generation of fluorophore is due entirely to the leak reaction between carrier and fuel which results in a linear production regime at short times where carrier and fuel concentrations do not change appreciably. After sink is depleted, no thresholding reaction is present to quench amplification and autocatalysis dominates the system. This timescale would allow enough time to build and trigger a waveguide before the leak reaction would trigger full activation, allowing a proof-of-concept demonstration of the system.

### 3.3. Delayed triggering of autocatalysis

**Figure 8.**
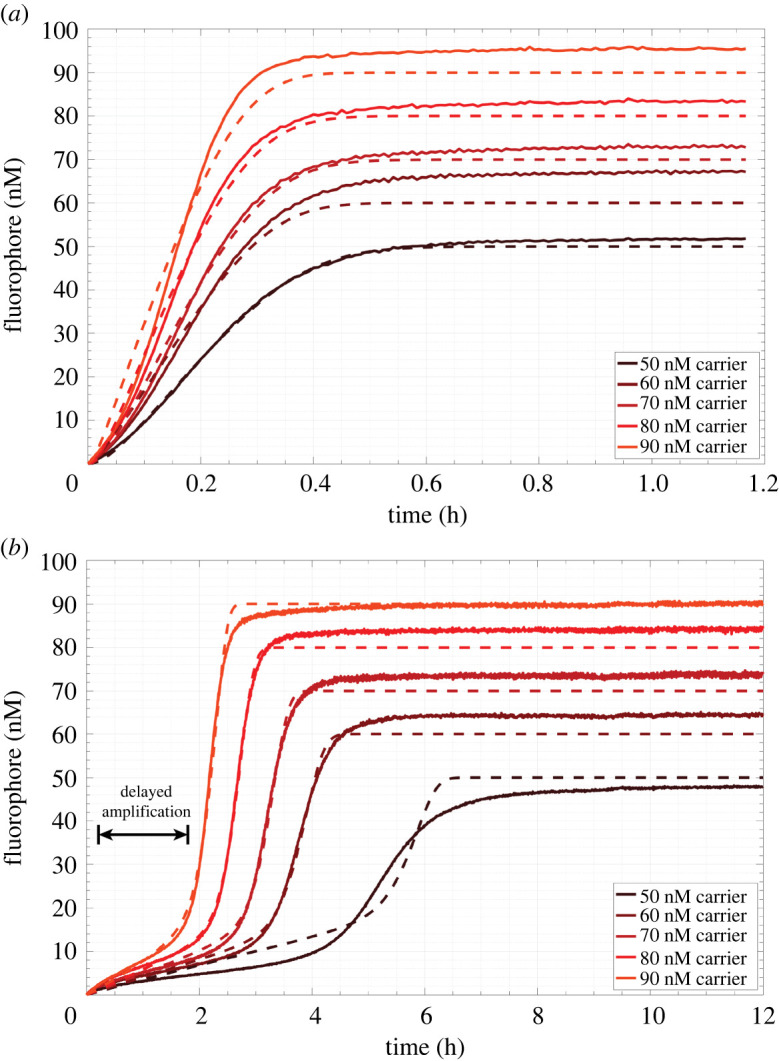
Kinetics of well-mixed autocatalytic reactions in experiments measured by changes in fluorescence. (*a*) Autocatalysis without thresholding by sink complex and (*b*) autocatalysis in the presence of a thresholding reaction driven by a 50 nM sink initial condition. Solid lines, experimentally measured concentration profiles; dashed lines, least-squares fit of a reaction model to experimental results.

**Figure 9.**
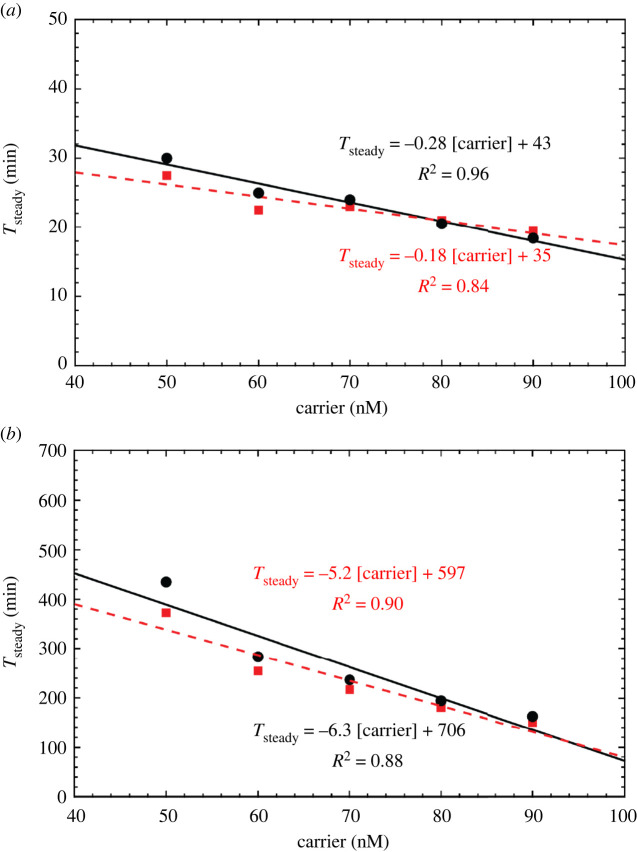
Equilibrium times for well-mixed autocatalysis: (*a*) without thresholding and (*b*) with 50 nM sink. Black circles are experimentally measured equilibrium times. Red squares indicate equilibrium times predicted by fitting the well-mixed model to experimental data. The solid black line is the linear least-squares fit to the experimental equilibrium times (black circles). The red dashed line is the linear least-squares fit to the equilibrium times predicted by the model (red squares).

We next sought to test in well-mixed experiments whether it was possible to trigger the circuit by adding a stimulus of signal while it was held in its lag phase by the sink reaction. We first measured the time needed for the circuit to reach equilibrium because of spurious activation, i.e. when no initial signal stimulus was added and in the absence of sink, to compare those values with those predicted by our simulations. We mixed a range of carrier concentrations (50 nM to 90 nM) with 200 nM fuel and 150 nM reporter in the absence of sink. Reactions were run at 25°C in a standard reaction buffer (1× Tris-acetate-EDTA buffer with 12.5 mM Mg^2+^). The fluorescence intensity increase of each individual reaction was measured in a Strategene quantitative PCR machine (electronic supplementary material, S2). We calibrated and converted fluorescence intensity into fluorophore concentration using separate calibration wells which were also measured during the experiment (electronic supplementary material, S4). To enable kinetic measurements, we selected carrier concentrations lower than those used in spatial models to ensure that we did not miss significant reaction progress while aliquoting reactants and loading the qPCR. We defined reaction equilibrium as the time at which the flourophore concentration increased to 95% of the final mean concentration in the plateau of the kinetic trace. The final mean concentration was determined by averaging the last 15 measurements made during an experiment. Across all carrier concentrations, we observed that the time to reach equilibrium was under 40 min after the reaction was initiated by the addition of fuel (figure 8*a*). The equilibrium times for each reaction condition are listed in table 1. Additionally, the time needed to reach equilibrium decreased linearly with increasing carrier concentration. To measure the actual reaction rate constants for the circuit, we fitted the rates in an ODE model of the amplification circuit to the data using nonlinear least-squares regression for each carrier concentration condition (electronic supplementary material, S4 and S6, table S2). The magnitude of the fitted rate constants was consistent with the expected order of magnitude of the rate constants based on toehold length [49]. The expected equilibrium fluorophore concentration for each test condition was 50 nM, 60 nM, 70 nM, 80 nM and 90 nM. The differences between the predictions of the model and the measured kinetics are shown in figure 8*a*. The model was able to fit the measured times to reach equilibrium as a function of carrier concentration (figure 9*a*) to within 8 min. Across all conditions, the measured concentration of fluorophore was slightly greater than the expected equilibrium concentration predicted by the complete reaction of fuel and carrier. Deviations in measured equilibrium fluorophore concentrations from targeted concentrations may have been caused by inaccuracies in initial reactant concentrations introduced via pipetting error (i.e. adding slightly too much or too little reporter or output in reaction wells or calibration wells, respectively)*.* These inaccuracies could affect both the reactions and the calibration process. The equilibrium concentration deviations are within typical ranges reported in the literature for DNA strand displacement reactions measured via qPCR, which achieve ±10 nM of targeted equilibrium fluorophore concentrations [51,52].

**Table 1.** Measured equilibrium times for unthresholded and thresholded amplification.

	50 nM carrier	60 nM carrier	70 nM carrier	80 nM carrier	90 nM carrier
0 nM sink	30 min	25 min	24 min	21 min	19 min
50 nM sink	7.3 h	4.7 h	3.9 h	3.2 h	2.7 h
X-fold increase	15	11	9.8	9.4	8.8

Having determined the expected timescale for spurious activation of the unthresholded amplifier, we next tested whether the addition of sink would delay the onset of amplification and whether a thresholded amplifier could be held in an off state where it could be triggered by an input signal. Importantly, the shape of the fluorophore curve resulting from thresholded amplification should have a sigmoidal profile. The curve should have an initial period of sub-exponential growth where the threshold quenches autocatalysis by acting as a competitor, followed by autocatalysis after sink is depleted (figure 8*b*). Such behaviour would indicate that the circuit could eventually undergo exponential growth when a trigger is added, as the trigger would deplete the sink rapidly. This would then cause the circuit to transition from a lag phase to exponential growth. Conversely, an excess concentration of sink relative to the carrier concentration would prevent autocatalysis from occurring and the rate of output production would only be coupled to the bimolecular reaction of fuel and carrier, which would not result in a sigmoidal growth curve. To identify concentrations of sink and carrier where autocatalysis would be delayed but not entirely prevented, we repeated the experiments previously described under the same conditions but mixed 50 nM sink into each reaction well at the start of the experiment. The time needed to reach equilibrium increased for each reaction with respect to its corresponding process without sink. The minimum time to reach equilibrium was 2.7 h (for the highest carrier concentration) (table 1). Just as in the absence of sink, we observed a roughly linear relationship between the time needed to reach equilibrium and the initial carrier concentration in the presence of 50 nM sink (figure 9*b*). On average across carrier concentrations, the addition of 50 nM sink increased the time needed to reach equilibrium by a factor of 11 ± 3 (95% confidence interval).

**Figure 10.**
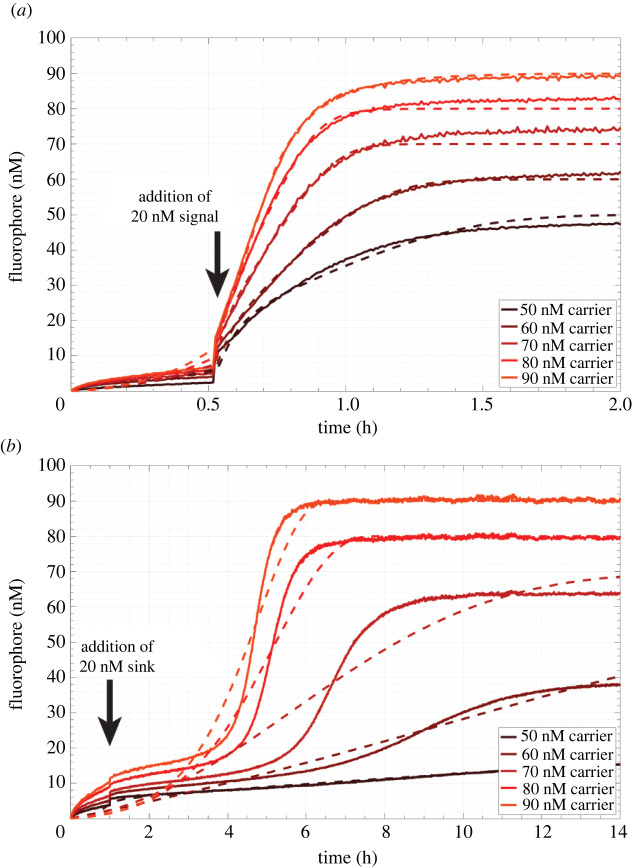
Perturbation of amplification during thresholding. Solid lines, experimental data; dashed lines, results of least-squares regression. (*a*) Delayed triggering of autocatalysis via addition of 20 nM signal 32 min after initiation of the experiment. (*b*) Extended delay of autocatalysis by the addition of 20 nM sink roughly 1 h after initiation of the reaction.

We then sought to trigger the circuit during its lag phase by adding signal to both verify that exponential amplification could occur and identify the size of the signal stimulus needed to induce exponential amplification. The experimental conditions were identical to those described previously. First, sink, fuel and reporter were each mixed together in five different reaction wells at final concentrations of 50 nM, 200 nM and 150 nM, respectively. Carrier was then added to each of the five reaction wells to final concentrations of 50 nM, 60 nM, 70 nM, 80 nM and 90 nM to initiate the reactions (figure 10*a*). We then waited 30 min, during which time we observed a slow and gradual increase of fluorophore concentration, consistent with the lag phase of the reaction. After 32 min, we added aliquots of signal corresponding to final concentrations of 20 nM to each of the reactions; we observed sharp increases in the fluorophore concentration curves, consistent with autocatalytic amplification. These reactions appeared to go to completion: for all carrier concentrations, the final concentration of fluorophore was within 7 nM of the concentration expected for complete reaction of carrier and fuel. A least-squares fit to the ODE model (figure 10*a*, dashed lines) was able to match the measured times needed to reach equilibrium for each of the carrier concentrations (electronic supplementary materials, S4).

To compare this result to the effect of further delaying autocatalysis by adding more sink, which should provide additional energy to suppress autocatalysis, we conducted the same experiment but added 20 nM of sink instead of 20 nM signal 1 h after initiating the reactions (figure 10*b*). The addition of 20 nM of sink increased the total concentration of sink to 70 nM, which should saturate 50 nM and 60 nM carrier concentrations and prevent amplification. The fluorescence curves for 60–90 nM carrier had a sigmoidal shape. At 50 nM carrier, we observed the slowest increase in fluorophore across all conditions and no visible inflection of the fluorescence curve, which indicated an absence of exponential growth and inhibition of autocatalysis. This condition did not reach equilibrium during the timescale of measurement suggesting that the circuit was saturated with sink. At 60 nM carrier, we observed a flattened sigmoidal curve indicating that a minimal degree of autocatalysis occurred during the reaction. For this condition, the equilibrium fluorophore concentration was below the expected concentration of 60 nM. As a result, the least-squares fit of the ODE model overpredicted the timescale of pre-exponential growth after the addition of 20 nM sink yielding a significant difference between inflection timepoints between the model and experimental curve. Only samples with carrier concentrations of 80 nM and 90 nM reached their targeted equilibrium concentrations over the timescale of measurement and had equilibrium times of 6.1 h and 5.5 h, respectively, which were both a factor of 2 greater than the equilibrium times attained by equivalent reactions in the presence of an initial concentration of 50 nM sink alone. Thus, at 80 nM and 90 nM carrier, the net addition of 70 nM sink mixed into to the circuit at different times before the onset of exponential growth could extend the lag phase.

### 3.4. Mitigation of spurious amplification resulting from leak reactions

**Figure 11.**
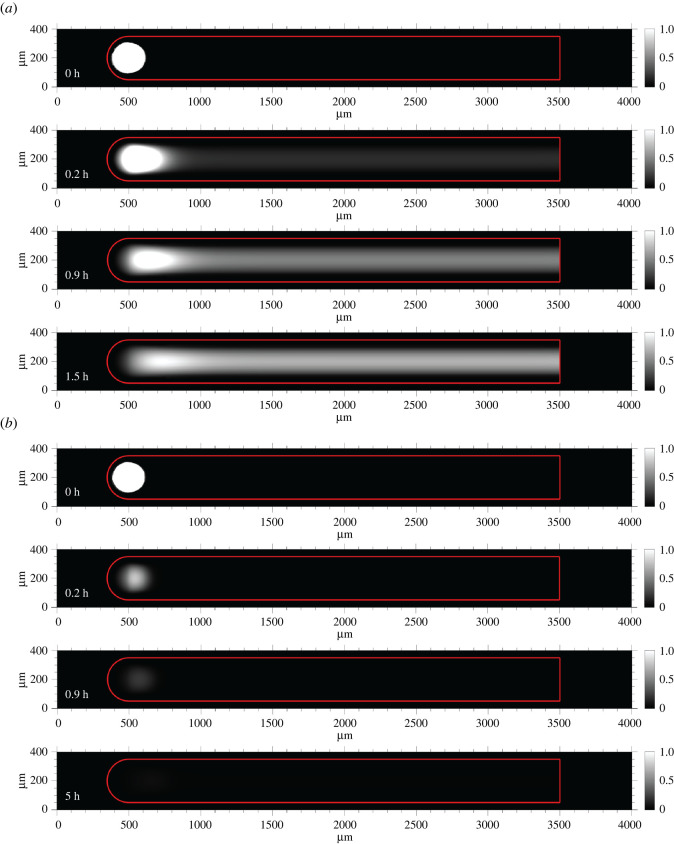
Predictions of a model of spatio-temporal wavefront propagation with non-idealized amplification, thresholding and fuel–carrier leak reactions. (*a*) Propagation failure and spurious waveguide activation without patterned sink due to fuel–carrier leakage. (*b*) Propagation failure with 35 nM sink patterned within the waveguide core. Surface plots show the signal concentration divided by the maximum signal concentration attained on the wavefront.

Having measured the rate constants of the designed and unintended leak reactions, we then modelled the reaction–diffusion waveguide using the strand displacement reactions in figure 6*a,b* (see electronic supplementary material, S3, for CRN implementation). The model initially used the same initial concentration conditions as those stated for the idealized spatial simulations where the carrier concentration was 270 nM; the initial concentration of fuel along the waveguide core was 270 nM. The bimolecular rate constants for the strand displacement reaction–diffusion system were based on the expected orders of magnitude which are functions of toehold size [49]. The results of the initial model are shown in figure 11. In the absence of any sink within the waveguide core, an initial wave of signal was observed at 12 min. The spurious generation of signal from the leak reaction between fuel and carrier within the body of the waveguide is evident by 1.5 h and grows to turn the whole waveguide on before the wavefront has arrived (figure 11*a*). When 35 nM of sink is sequestered within the waveguide core, we observed an initial pulse of signal at 12 min; the wave decayed away by 54 min and similarly failed to travel along the waveguide (figure 11*b*). As a result of these two particular failure modes, we hypothesized that the rate of amplification for the concentration conditions selected was not high enough to produce a wave that could overcome the energetic drain of the patterned sink and insulation.

**Figure 12.**
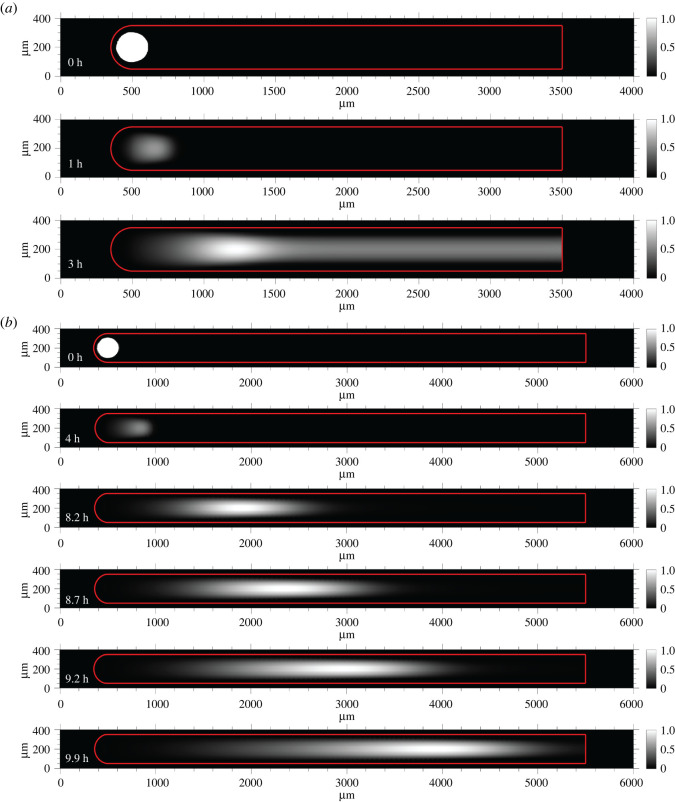
(*a*) Spurious waveguide activation during wavefront displacement due to carrier–fuel leak reaction. Initial concentrations of carrier, fuel and sink were 650 nM, 650 nM and 84.24 nM, respectively. (*b*) Travelling wave of signal along waveguide core when carrier, fuel and sink are patterned as linearly increasing gradients.

We next asked whether increasing the rate of amplification would overcome these limitations to enable wave propagation along the waveguide. To achieve this, we modelled a waveguide of the same geometry as the previous simulations. The initial concentrations of carrier, fuel and sink patterned in the waveguide were 650 nM, 650 nM and 84.24 nM; the ratios of carrier, fuel and sink were kept identical to the earlier simulations of the DNA reaction–diffusion waveguide. The concentration of sink in the insulation was increased to 1000 nM to prevent the wave from diffusing beyond the waveguide. At 1 h of simulated time, we observed a wave of signal that displaced roughly 100 µm from the starting position (figure 12*a*). However, at 3 h of simulated time, the entire waveguide became active and produced signal, which corresponded with the complete consumption of sink, before the wavefront had travelled down the waveguide. Based on this result, we proposed that patterning the reactants as linearly increasing gradients along the length of the waveguide would ensure that sink was not depleted during the time it took for the wavefront to arrive at a particular location on the waveguide. Additionally, we hypothesized that a linearly increasing gradient would result in a sufficient concentration of carrier and fuel at a location ahead of the wavefront so that the wave could continue to propagate and not decay in magnitude or slow its rate of displacement due to the absence of carrier and fuel. For example, we modified the simulation so that the concentration of carrier increased linearly from 780 nM to 1013 nM along the length of the waveguide. The forms of the gradients of fuel, sink and carrier employed in the model are plotted in the electronic supplementary material, figure S5. The starting concentration of signal in the trigger domain was increased to 220 nM, and the concentration of sink in the insulation was increased 1500 nM. We also increased the length of the waveguide to 5000 µm to study the evolution of the wavefront over a longer distance. When the simulation employed linearly increasing gradients of reactants, a wave of signal propagated along the length of the waveguide (figure 12*b*). Analysis of the wave displacement versus time indicated that the system minimally achieved directed transport, where *α* = 2.34 ± 0.04 (95% confidence interval) (electronic supplementary material, figures S6 and S9c). These results suggest that patterning sink, fuel and carrier as linear gradients within the waveguide would serve as an effective strategy for suppressing the autocatalytic leak reaction in single usage experiments.

Moving beyond this analysis, we asked whether there are molecular protection strategies that further mitigate the risk of spurious amplification during waveguide construction (i.e. during photolithographic processes) by keeping the carrier inactive until its activation is triggered. One protection strategy that could make carrier inactive and prevent leakage is photoprotection using photocleavable 1-(2-nitrophenyl) ethyl linkers that can be incorporated into the phosphodiester backbone of synthetic oligonucleotides.

Such photoprotection of DNA strand displacement reactions using nitrobenzyl chemistries has been demonstrated [53,54]. UV light applied along the waveguide might be used to photo-deprotect cross-linked carrier right before its use. During testing of the system, we also envision applying this activation just ahead of the signal as it travels along the length of the wire. Control by photoactivation would minimize the amount of time during which active carrier and fuel could react spuriously before the arrival of a wavefront.

We also hypothesized that the dominant mechanism of carrier–fuel leakage occurred through hybridization of the fuel strand to transiently exposed nucleotides at the ends of the carrier duplex, whereby fuel nucleated to exposed carrier bases and then branch migrated to displace signal. In this case, the addition of seven nucleotide length clamp domains to each end of the carrier complex would slow the rate of the carrier–fuel leak by occluding the ends of the carrier duplex to prevent invasion by the fuel (electronic supplementary material, figure S7). However, these clamping domains would also prevent the desired reaction between carrier and signal from occurring during waveguide operation and would require a removal mechanism to yield an active form of the carrier. UV photocleavable spacers connecting the clamp domains to the 5′ end of signal and the 3′ end of output would provide a mechanism for cleaving the clamps to allow autocatalysis to occur at the designed time when the waveguide is exposed to UV light (see supplementary material, S6 Results and Discussion, for photoprotection mechanism).

We experimentally tested whether a clamped carrier possessing seven nucleotide length clamp domains would block the leak reaction induced by fuel (see electronic supplementary material, S6 Results and Discussion, for experimental details). However, we observed that incubation of clamped carrier species with fuel failed to prevent the leak reaction in well-mixed experiments (electronic supplementary material, figure S8). The persistence of the carrier–fuel leak reaction and the size of the fitted leak rate constant indicated that the protection strategy for the duplex ends was not effective in preventing the invasion of fuel strand. This suggested that the dominant mechanism occurring during the leak reaction was fuel hybridization to transiently exposed bases at the nick site within carrier. The proposed hybridization occurred between the 3′ end of signal and the 5′-most nucleotide of output bound to the bottom strand of carrier (Carrier_b_); Lysne *et al*. [42] independently identified the same leak mechanism involving the carrier nick site. One possible way of occluding the nick to prevent the leak reaction would be to introduce a non-canonical photocleavable attachment between the 5′ end of the last output nucleotide bound to Carrier_B_ and the 3′ carbon of the first signal nucleotide hybridized to Carrier_B_. However, further study of this strategy would be needed; to our knowledge, the use of such photosensitive modifications within strand displacement networks has not yet been demonstrated.

## 4. Discussion

In this study, we use computational analyses and measure the kinetics of reactions in well-mixed solution to support the idea that super-diffusive propagation of chemical waves using DNA strand displacement amplification should be feasible over length scales of hundreds of micrometres using concentration ranges of oligonucleotides commonly used in strand displacement processes [55–57]. The use of thresholding reactions provides a way of mitigating deleterious fuel–carrier side reactions that might otherwise trigger spurious amplification. The integration of strand displacement waveguides into existing classes of DNA-based soft materials might enable chemical signal transmission within biomaterials and between separated devices over timescales orders of magnitude shorter than what could be achieved with diffusion alone. Moreover, the ability to combine different sets of stimuli using wires will provide control over where and how chemical information is distributed within a biomaterial, enabling coordinated responses to complex sets of environmental cues [58–60].

To implement a full hydrogel waveguide experimentally, further investigation of orthogonal microfabrication methods and DNA-compatible nucleic acid photochemistries is required. Photolithographic techniques offer the capability of precisely designing patterned biomaterials at biologically relevant size scales within a controlled environment, which is a requirement for strand displacement reactions due to temperature and pH sensitivity. The placement of oligonucleotide patterns within a substrate via photopolymerization to accommodate subsequent photo-directed release or activation of cross-linked species serves as a proxy for spatial biomolecular stimuli that might eventually induce activation of a waveguide and would enable precise spatio-temporal activation of these architectures for further study and optimization.

## Data Availability

Datasets and MATLAB code supporting this article are available in the Dryad Digital Repository [61]. Electronic supplementary material is available online [62].

## Footnotes

1 An important distinction exists between the reaction–diffusion waveguides we propose and those used for propagating sound or electromagnetic waves. While both systems direct waves by restricting energy transmission in a specific direction, our reaction–diffusion system achieves propagation by exponentially amplifying its signal to maintain its concentration instead of minimizing energy loss.
